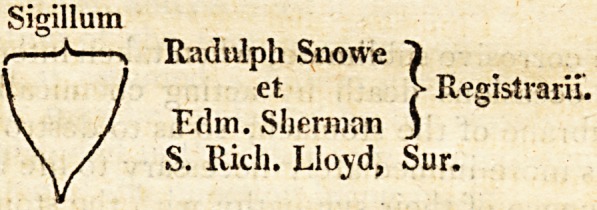# Collectanea Medica

**Published:** 1812-08

**Authors:** 


### COLLECTANEA MEDICA,

CONSISTING OF

### ANECDOTES, FACTS, EXTRACTS, ILLUSTRATIONS, QUERIES, SUGGESTIONS, &c.

RELATING TO THE

*History or the Art of Medicine, and the Auxiliary Sciences.*

---

*Further Experiments and Observations on the Action of Poisons on the Animal System. By* B. C. BRODIE, Esq. F.R.S. *From the Philosophical Transactions. Read before the Royal Society, Feb.* 27, 1812.

I. SINCE I had the honor of communicating to the Royal Society some observations on the action of certain poisons on the animal system, I have been engaged in the further prosecution of this inquiry. Besides some additional experiments on vegetable poisons, I have instituted several with a view to explain the effects of some of the more powerful poisons of the mineral kingdom. The former correspond in their results so nearly with those which are already before the public, that, in the present communication, I shall confine myself to those which appear to be of some importance, as they more particularly confirm my former conclusions respecting the recovery of animals apparently dead, where the cause of death operates exclusively on the nervous system. In my experiments on mineral poisons, I have found some circumstances wherein their effects differ from those of vegetable poisons, and of these I shall give a more particular account. Whatever may be the value of the observations themselves, the subject must be allowed to be one that is deserving of investigation, as it does not appear unreasonable to expect that such investigation may hereafter lead to some improvements in the healing art. This consideration, I should hope, will be regarded as a sufficient apology for my pursuing a mode of inquiry by means of experiments on brute animals, of which we might well question the propriety, if no other purpose were to be answered by it than the gratification of curiosity.

In my former communication on this subject, I entered into a detailed account of the majority of my experiments. This I conceived necessary, because in the outset of the inquiry I had been led to expect that even the same poison might not always operate precisely in the same manner; but I have since had abundant proof, that in essential circumstances there is but little variety in the effects produced by poisons of any description, when employed on animals of the same, or even of different, species, beyond what may be referred to the difference in the quantity, or mode of application of the poison, or of the age and power of the animal. This will explain the reason of my not detailing, in the present communication, so many of the individual experiments from which my conclusions are drawn, as in the former; at the same time I have not been less careful to avoid drawing general conclusions from only a limited number of facts. Should these conclusions prove fewer, and of less importance than might be expected, such defects will, I trust, be regarded with indulgence; at least by those, who are aware of the difficulty of conducting a series of physiological experiments; of the time which they necessarily occupy; of the numerous sources of fallacy and failure which exist; and of the laborious attention to the minutest circumstances, which is in consequence necessary in order to avoid being led into error.

II. *Experiments with the Woorara.*—In a former experiment, I succeeded in recovering an animal, which was apparently dead from the influence of the essential oil of bitter almonds, by continuing respiration artificially until the impression of the poison upon the brain had ceased; but a similar experiment on an animal under the influence of the woorara was not attended with the same success. Some circumstances led me to believe, that the result of the experiment with the woorara might have been different, if it had been made with certain precautions; but I was unable at that time to repeat it, in consequence of my stock of the poison being exhausted. I have since, however, been able to procure a fresh supply, and I shall relate two experiments which I have made with it. In one of these, an animal apparently dead from the woorara, was made to recover, notwithstanding the functions of the brain appeared to be wholly suspended for a very long period of time; in the other, though ultimate recovery did not take place, the circulation was maintained for several hours after the brain had ceased to perform its office.

*Experiment* 1. Some woorara was inserted into a wound in a young cat. She became affected by it in a few minutes, and lay in a drowsy and half-sensible state, in which she continued at the end of an hour and fifteen minutes, when the application of the poison was repeated. In four minutes after the second application, respiration entirely ceased, and the animal appeared to be dead; but the heart was still felt acting about one hundred and forty times in a minute. She was placed in a temperament of 85 of Fahrenheit's thermometer, meter, and the lungs were artificially inflated about forty times in a minute.

The heart continued acting regularly.

When the artificial respiration had been kept up for forty minutes, the pupils of the eyes were observed to contract and dilate on the increase or diminution of light; saliva had flowed from the mouth, and a small quantity of tears was collected between the eye and eye-lids; but the animal continued perfectly motionless and insensible.

At the end of an hour and forty minutes, from the same period, there were slight involuntary contractions of the muscles, and every now and then there was an effort to breathe. The involuntary motions continued, and the efforts to breathe became more frequent. At the end of another hour, the animal, for the first time, gave some signs of sensibility when roused, and made spontaneous efforts to breathe twenty-two times in a minute. The artificial respiration was discontinued. She lay, as if in a state of profound sleep, for forty minutes, when she suddenly awoke and walked away. On the following day she appeared slightly indisposed; but she gradually recovered, and is at this time still alive and in health.

*Experiment* 2. Some woorara was applied to a wound in a rabbit. The animal was apparently dead in four minutes after the application of the poison; but the heart continued acting. He was placed in a temperature of 90°, and the lungs were artificially inflated. The heart continued to act about one hundred and fifty times in a minute. For more than three hours the pulse was strong and regular; after this, it became feeble and irregular, and at the end of another hour the circulation had entirely ceased. During this time there was no appearance of returning sensibility.

The circulation of the blood may be maintained in an animal from whom the brain has been removed for a considerable, but not for an unlimited, time. We may conclude, that in the last of these experiments the animal did not recover, because the influence of the poison continued beyond the time during which the circulation may be maintained without the brain.

III. *On the Effects of Arsenic.*—When an animal is killed by arsenic taken internally, the stomach is found bearing marks of inflammation; and it is a very general opinion, 1, that this inflammation is the cause of death: 2, that it is the consequence of the actual contact of the arsenic with the internal coat of the stomach. But in several cases I have found the inflammation of the stomach so slight, that on a superficial examination it might have been easily overlooked; and in in most of my experiments with this poison, death has taken place in too short a time for it to be considered as the result of inflammation: and hence we may conclude, that the first of these opinions is incorrect; at least as a general proposition.

Many circumstances conspire to show that the second of these opinions also is unfounded.

In whatever way the poison is administered, the inflammation is confined to the stomach and intestines; I have never seen any appearance of it in the pharynx or œsophagus.

Mr. Home informed me, that in an experiment made by Mr. Hunter and himself, in which arsenic was applied to a wound in a dog, the animal died in twenty-four hours, and the stomach was found to be considerably inflamed.

I repeated this experiment several times, taking the precaution always of applying a bandage to prevent the animal licking the wound. The result was, that the inflammation of the stomach was commonly more violent and more immediate, than when the poison was administered internally, and that it preceded any appearance of inflammation of the wound.* Some experiments are already before the public, which led me to conclude that vegetable poisons, when applied to wounded surfaces, affect the system by passing into the circulation through the divided veins. From this analogy, and from all the circumstances just mentioned, it may be inferred that arsenic, in whatever way it is administered, does not produce its effects even on the stomach until it is carried into the blood.

But the blood is not necessary to life, except so far as a constant supply of it is necessary for the maintenance of the functions of the vital organs. The next object of inquiry therefore is, when arsenic has entered the circulation, on what organs does it operate, so as to occasion death?

When arsenic is applied to an ulcerated surface, it produces a slough, not by acting chemically, like caustics in general, but by destroying the vitality of the part to which it is applied, independently of chemical action. This led

---

* Since the greater part of my experiments on this subject were made, I have seen an account of an inaugural Dissertation on the Effects of Arsenic, by Dr. Jaëger, of Stuttgard. Dr. Jaëger has come to conclusions similar to those above stated, that in an animal killed by arsenic, the inflammation of the stomach is not the cause of death, and that the poison does not produce its fatal effects until it has entered the circulation. I have to regret that I have had no opportunity of seeing the original of this Dissertation.

me me at first to suppose, that when arsenic has passed into the circulation, death is the consequence, not so much of the poison disturbing the functions of any particular organ, as of its destroying at once the vitality of every part of the system. The following circumstances, however, seem to show that this opinion is erroneous. In an animal under the full influence of arsenic, even to the instant of death, some of the secretions, as those of the kidneys, stomach, and intestines, continue to take place in large quantity; and the muscles are capable of being excited, after death, to distinct and powerful contractions by means of the Voltaic battery.

*Experiment* 3. Seven grains of the white oxide of arsenic were applied to a wound in the back of a rabbit.

In a few minutes he was languid, and the respirations were small and frequent. The pulse was feeble, and after a little time could not be felt. The hind legs became paralysed.* He grew insensible, and lay motionless, but with occasional convulsions. At the end of fifty-three minutes from the time of the arsenic being applied, he was apparently dead; but on opening the thorax, the heart was found still acting, though very slowly and feebly. A tube was introduced into the trachea, and the lungs were artificially inflated; but this appeared to have no effect in prolonging the heart's action. On dissection, the inner membrane of the stomach was found slightly inflamed.

*Experiment* 4. Two drams of arsenic acid dissolved in six ounces of water were injected into the stomach of a dog, by means of a tube of elastic gum, passed down the œsophagus. In three minutes he vomited a small quantity of mucus, and this occurred again several times. The pulse became less frequent, and occasionally intermitted. At the end of thirty-five minutes the hind legs were paralysed, and he lay in a half-sensible state. At the end of forty-five minutes he was less sensible; the pupils of the eyes were dilated; the pulse had fallen from 140 to 70 in a minute, and the intermissions

* I have observed, that where the functions of the brain are disturbed, paralysis first takes place in the muscles of the hind legs; afterwards in those of the trunk and fore legs; and last of all in the muscles of the ears and face. These facts seem to show that the influence of the brain, like that of the heart, is not propagated with the same facility to the distant as to the near organs; and this is further confirmed by cases of disease which occasionally occur, in which, although the paralysis is confined to the lower half of the body, the morbid appearances met with on dissection are entirely confined to the brain.

were were frequent. After this, he became quite insensible; convulsions took place, and at the end of fifty minutes, from the beginning of the experiment, he died. On opening the thorax, immediately after death, tremulous contractions of the heart were observed; but not sufficient to maintain the circulation. The stomach and intestines contained a large quantity of mucous fluid, and their internal membrane was highly inflamed.

These experiments were repeated, and the results, in all essential circumstances, were the same. The symptoms produced were, 1. Paralysis of the hind legs, and afterwards of the other parts of the body; convulsions; dilatation of the pupils of the eyes; insensibility; all of which indicate disturbance of the functions of the brain. 2. A feeble, slow, intermitting pulse, indicating disturbance of the functions of the heart. Where the heart has continued to act after apparent death, I have never, in any one instance, been able to prolong its action by means of artificial respiration. 3. Pain in the region of the abdomen; preternatural secretion of mucus from the alimentary canal; sickness and vomiting in those animals which are capable of vomiting; symptoms which arise from the action of the poison on the stomach and intestines. There is no difference in the effects of arsenic, whether it is employed in the form of white oxide, or of arsenic acid, except that the latter is a more active preparation. When arsenic is applied to a wound, the symptoms take place sooner than when it is given internally; but their nature is the same.

The symptoms produced by arsenic may be referred to the influence of the poison on the nervous system, the heart,* and the alimentary canal. As of these the two former only are concerned in those functions, which are di-

* When I say that a poison acts on the heart, I do not mean to imply that it necessarily must act directly on the muscular fibres of that organ. It is highly probable, that the heart is affected only through the medium of its nerves; but the affection of the heart is so far independent of the affection of the nervous system generally, that the circulation may cease although the functions of the brain are not suspended, and the functions of the brain may be wholly suspended without the circulation being at all disturbed. In proof of the first of these propositions, I may refer to my former experiments on the upas antiar, in which the sensibility of the animal continued to the very instant of death; and respiration, which is under the influence of the brain, continued even after the heart had ceased to act. In proof of the second, I may refer, among many others, to the experiments detailed in the Croonian Lecture for 1810.

rectly necessary to life, and as the alimentary canal is often affected only in a slight degree, we must consider the affection of the heart and nervous system as being the immediate cause of death.

In every experiment which I have made with arsenic, there were evident marks of the influence of the poison on all the organs which have been mentioned; but they were not in all cases affected in the same relative degree. In the dog, the affection of the heart appeared to predominate over that of the brain, and on examining the thorax, immediately after death, this organ was found to have ceased acting and in a distended state. In the rabbit, the affection of the brain appeared to predominate over that of the heart, and the latter was usually found acting slowly and feebly, after the functions of the brain had entirely ceased. In the rabbit, the effects of the arsenic on the stomach and intestines were usually less than in carnivorous animals.

The action of arsenic on the system is less simple than that of the majority of vegetable poisons. As it acts on different organs, it occasions different orders of symptoms, and as the affection of one or another organ predominates, so there is some variety in the symptoms produced even in individual animals of the same species.

In animals killed by arsenic the blood is usually found fluid in the heart and vessels after death; but otherwise all the morbid appearances met with on dissection are confined to the stomach and intestines. As this is the case, and as the affection of these organs occasions remarkable symptoms, it may be right to mention the result of my observations on this subject.

In many cases where death takes place, there is only a very slight degree of inflammation in the alimentary canal: in other cases the inflammation is considerable. It generally begins very soon after the poison is administered, and appears greater or less according to the time which elapses before the animal dies. Under the same circumstances, it is less in graminivorous than in carnivorous animals. The inflammation is greatest in the stomach and rectum, but it usually extends also over the whole intestine. I have never observed inflammation of the œsophagus. The inflammation is greater in degree, and more speedy in taking place, when arsenic is applied to a wound, than when it is taken into the stomach. The inflamed parts are in general universally red, at other times they are red only in spots. The principal vessels leading to the stomach and intestines are turgid with blood; but the inflammation is usually confined to the mucous membrane of these viscera, which assumes a florid red color, becomes soft and pulpy, and is separable without much difficulty from the cellular coat, which has its natural appearance. In some instances there are small spots of extravasated blood on the inner surface of the mucous membrane, or between it and the cellular coat, and this occurs independently of vomiting. I have never, in any of my experiments, found ulceration or sloughing of the stomach or intestine; but if the animal survives for a certain length of time, after the inflammation has begun, it is reasonable to conclude that it may terminate in one or other of these ways.

I am disposed to believe that sloughing is very seldom, if ever, the direct consequence of the application of arsenic to the stomach or intestines. Arsenic applied to an ulcer will occasion a slough; but its action in doing this is very slow. When I have applied the white oxide of arsenic to a wound, though the animal has sometimes lived three or four hours afterwards, and though violent inflammation has taken place in the stomach and intestines, I have never seen any preternatural appearance in the part to which it was applied, except a slight effusion of serum into the cellular membrane. Arsenic speedily produces a very copious secretion of mucus and watery fluid from the stomach and intestines, which separates it from actual contact with the inner surface of these organs, even though taken in large quantity and in substance; and in animals which are capable of vomiting, by much the greater part is rejected from the stomach very soon after it has been taken in. Hence, though a few particles of arsenic are sometimes found entangled in the mucus, or in the coagulum of extravasated blood, and adhering to the inner surface of the stomach, I have never seen it in such a quantity as might be supposed capable of producing a slough. In one instance, where a dog had swallowed a large quantity of arsenic in substance, a brown spot, about an inch in diameter, was observed after death on the inner surface of the cardiac extremity of the stomach, having so much of the appearance of a slough, that at first I had no doubt of it being so; but on examination this proved to be only a thin layer of dark-colored coagulum of blood, adhering very firmly to the surface of the mucous membrane, and having a few particles of arsenic entangled in it. On removing this the mucous membrane still appeared of a dark color; but this was also found to arise from a thin layer of coagulum of blood between it and the cellular coat. The mucous membrane itself was inflamed; but otherwise in a natural state. I have observed a similar appearance, but occupying a less extent of surface, several times. In the the Hunterian Museum there is a human stomach, which was preserved to show what was considered as a slough produced by the action of arsenic. On examining this preparation, I found that the dark-colored spot, which had been supposed to be a slough, was precisely of the same nature with that just described.

Although the affection of the stomach and intestines from arsenic is not the cause of death, under ordinary circumstances, it is reasonable to conclude that it may be so in some instances, if the animal survives the effects produced on the organs more immediately necessary to life. Mr. Henry Earle informed me of an instance, in which this appeared to be the case. A woman in St. Bartholomew's hospital, who had taken arsenic, recovered of the immediate symptoms, but died at the end of four or five days. On examination after death, extensive ulcerations were found of the mucous membrane of the stomach and intestines, which we can hardly doubt to have been the cause of death.

It is an important matter of inquiry, as connected with judicial medicine, how far may the examination of the body after death, enable us to decide whether an animal has died of the effects of arsenic? On this subject, however, I have only a few remarks to make.

The inflammation from arsenic, occupying in general the whole of the stomach and intestine, is more extensive than that from any other poison with which I am acquainted. It does not affect the pharynx or œsophagus, and this circumstance distinguishes it from the inflammation which is occasioned by the actual contact of irritating applications.

But little in general is to be learnt from the examination of the contents of the stomach after death. When arsenic has been taken in substance, small particles of it are frequently found entangled in the mucus, or in the extravasated blood; but where this was not the case, I have never known, in an animal that was capable of vomiting, that arsenic could be detected in the contents of the stomach after death, though examined by the most accurate chemical tests. As some substances, when taken internally, are separated from the blood very soon afterwards with the urine, I thought it probable that arsenic might be separated with the urine also; but Mr. Brande (to whom I am indebted for assistance on this, as well as on many other occasions) could never detect the smallest trace of arsenic in it.

IV. *Experiments with the Muriate of Barytes.*—When barytes is taken into the stomach, or applied to a wound, it is capable of destroying life; but when in its uncombined state its action is very slow. The muriate of barytes, which is much more soluble than the pure earth, is (probably on this account) a much more active poison.

*Experiment* 5. Ten grains of muriate of barytes rubbed very fine, and moistened with two drops of water, were applied to two wounds in the thigh and side of a rabbit. In four minutes he was evidently under the influence of the poison. In a short time he became giddy; then his hind legs were paralysed; and he gradually fell into a state of insensibility, with dilated pupils, and lay in general motionless, but with occasional convulsions. The pulse beat 150 in a minute, but feeble, and it occasionally intermitted. He was apparently dead in twenty minutes from the application of the poison; but, on opening the chest, the heart was found still acting, and nearly three minutes elapsed before its action had entirely ceased.

*Experiment* 6. An ounce and a half of saturated solution of muriate of barytes was injected into the stomach of a full grown cat, by means of an elastic gum tube. In a few minutes it operated as an emetic. The animal became giddy, afterwards insensible, and lay with dilated pupils, in general motionless, but with occasional convulsions. At the end of sixty-five minutes from the beginning of the experiment, he was apparently dead; but the heart was still felt through the ribs acting one hundred times in a minute. A tube was introduced into the trachea, and the lungs were inflated about thirty-six times in a minute; but the pulse sunk notwithstanding, and at the end of seven minutes the circulation had entirely ceased.

From these experiments I was led to conclude that the principal action of the muriate of barytes is on the brain; but in the first the pulse was feeble and intermitting; in the second, although the artificial respiration was made with the greatest care, the circulation could not be maintained more than a few minutes. These circumstances led me to suspect, that, although this poison operates principally on the brain, it operates in some degree on the heart also. Further experiments confirmed this suspicion. In some of them the pulse soon became so feeble, that it could be scarcely felt; and its intermissions were more frequent; but in all cases the heart continued to act after respiration had ceased; and the cessation of the functions of the brain was therefore always the immediate cause of death. When I employed artificial respiration, after death had apparently taken place, I seldom was able to prolong the heart's action beyond a few minutes. In one case only it was maintained for three quarters of an hour. I never by these means succeeded in restoring the animal animal to life, although the experiments were made with the greatest care, and in a warm temperature. In some instances, after the artificial respiration had been kept up for some time, there were signs of the functions of the brain being in some degree restored; but the pulse, notwithstanding, continued to diminish in strength and frequency, and ultimately ceased. I shall detail one of these experiments, as it serves to illustrate the double action of this poison on the nervous and vascular systems.

*Experiment* 7. Some muriate of barytes was applied to a wound in the side of a rabbit. The usual symptoms took place, and at the end of an hour the animal was apparently dead; but the heart still continued to contract. He was placed in a temperature of 80°, and a tube being introduced into the nostril, the lungs were artificially inflated about thirty-six times in a minute.

When the artificial respiration had been maintained for four minutes, he appeared to be recovering; he breathed voluntarily one hundred times in a minute, and shewed signs of sensibility. The artificial respiration was discontinued. The voluntary respiration continued about nine minutes, when it had ceased, and the animal was again apparently dead, but the pulse continued strong and frequent. The lungs were again artificially inflated. At the end of four minutes the animal once more breathed voluntarily one hundred times in a minute, and repeatedly moved his limbs and eye-lids. The pulse became slower and more feeble.

In a few minutes the voluntary respiration again ceased, and the artificial respiration was resumed. The pulse had fallen to one hundred, and was feeble. The animal again breathed voluntarily; but he ceased to do so at the end of five minutes. The lungs were inflated as before, but he did not give any signs of life, nor was the pulse felt afterwards. On opening the thorax, the heart was found to have entirely ceased acting.

A probe having been introduced into the spinal marrow, it was found that by means of the Voltaic battery, powerful contractions might be excited, not only of the voluntary muscles, but also of the heart and intestines; from which it may be inferred, that the muriate of barytes, like arsenic, affects the circulation by rendering the heart insensible to the stimulus of the blood, and not by destroying altogether the power of muscular contraction.

The muriate of barytes affects the stomach, but in a less degree than arsenic. It operates as an emetic in animals that are capable of vomiting, but sooner when taken internally, than when applied to a wound. In general, but not constantly, stantly, there are marks of inflammation of the inner membrane of the stomach, but not of the intestine. In many instances there is a thin layer of dark colored coagulum of blood lining the whole inner surface of the stomach, and adhering very closely to it, so as to have a good deal of the appearance of a slough; and this is independent of vomiting, as, where I met with it, it occurred in rabbits.

The same circumstances, from which it may be inferred that arsenic does not produce its deleterious effects until it has passed into the same circulation, lead to the same conclusion with regard to the muriate of barytes.

V. *On the Effects of the Emetic Tartar.*—The effects of the emetic tartar so much resemble those of arsenic and of muriate of barytes in essential circumstances, that it would be needless to enter into a detail of the individual experiments made with this poison.

When applied to a wound, in animals which are capable of vomiting, it usually, but not constantly, operates very speedily as an emetic; otherwise I have found no material difference in the symptoms produced in the different species of animals, which I have been in the habit of employing as the subjects of experiment. The symptoms are paralysis, drowsiness, and at last complete insensibility; the pulse becomes feeble; the heart continues to act after apparent death; its action may be maintained by means of artificial respiration, but never for a longer period than a few minutes: so that it appears that this poison acts on the heart as well as on the brain, but that its principal action is on the latter. Both the voluntary and involuntary muscles may be made to contract after death, by means of Voltaic electricity. The stomach sometimes bears the marks of inflammation, but at other times it has its natural appearance. I have never seen any appearance of inflammation of the intestines. The length of time which elapses from the application of the poison, to the death of the animal, varies. In some instances it is not more than three quarters of an hour; but in others it is two or three hours, or even longer.

When a solution of emetic tartar was injected into the stomach of a rabit, the same symptoms took place as when it was applied to a wound.

VI. *On the Effects of the Corrosive Sublimate.*—When this poison is taken internally in very small and repeated doses, it is absorbed into the circulation, and produces on the system those peculiar effects which are produced by other preparations of mercury. If it passes into the circulation in larger quantity, it excites inflammation of some part of the alimentary canal, the termination of which may vary accordingly as it exists in a greater or less degree. When taken in a larger quantity still, it occasions death in a very short space of time. I had found, that if applied to a wounded surface, it produced a slough of the part to which it was applied, without occasioning any affection of the general system. This led me to conclude that the effects of it, taken internally and in a large quantity, depended on its local action on the stomach, and were not connected with the absorption of it into the circulation. The following experiments appear to confirm this opinion.

*Experiment* 8. Six grains of corrosive sublimate, dissolved in six drams of distilled water, were injected into the stomach of a rabbit, by means of an elastic gum tube. No immediate symptoms followed the injection; the animal made no expression of pain, but in three minutes he became insensible, was convulsed, and in four minutes and a half from the time of the injection being made, he died. Tremulous contractions of the voluntary muscles continued for some time afterwards. On opening the thorax, the heart was found to have entirely ceased acting, and the blood in the cavities of the left side was of a scarlet color. The stomach was much distended. The pyloric and cardiac portions were separated from each other by a strong muscular contraction. The contents of the former were firm and solid, and in every respect resembled the usual contents of the stomach; while those of the cardiac portion consisted of the food of the animal much diluted by fluid; so that the solution which had been injected, appeared to be confined to the cardiac portion of the stomach, and to be prevented entering the pyloric portion by the muscular contraction in the centre.

In the pyloric portion of the stomach the mucous membrane had its natural appearance; but in the cardiac portion it was of a dark grey color, was readily torn and peeled off, and in some parts its texture was completely destroyed, so that it appeared like a pulp, on removing which the muscular and peritoneal coats were exposed.

The repetition of the experiment was attended with similar results. The alteration of the texture of the internal membrane appears to have been occasioned by its being chemically acted on by the corrosive sublimate injected into it. When the injection is made into the stomach of a dead rabbit, precisely the same effects are produced, except that, as the middle contraction is here wanting, the appearances are not confined in the same degree to the cardiac portion.

*Experiment* 9. A scruple of corrosive sublimate, dissolved in six drams of distilled water, was injected into the stomach stomach of a full grown cat. For the first five minutes no symptoms were produced. After this, the poison operated twice as an emetic. The animal appeared restless, and made expression of pain in the abdomen. He gradually became insensible, and lay on one side motionless, with the pupils of the eyes dilated. The respiration was laborious, and the pulse could not be felt. Twenty-five minutes after the poison was injected there was a convulsive motion of the voluntary muscles, and death ensued. On opening the thorax immediately afterwards, the heart was seen still contracting, but very feebly.

The stomach was found perfectly empty and contracted. The mucous membrane was every where of a dark grey color. It had lost its natural texture, and was readily torn and separated from the muscular coat. The internal membrane of the duodenum had a similar appearance, but in a less degree, for nearly three inches from the pylorus. In the situation of the pylorus, the effects of the poison were less apparent than in any other part.

The particular state of the internal membrane of the stomach, in this experiment as well as in the last, appears to have been occasioned by the chemical action of the poison on it. When I injected a solution of corrosive sublimate into the stomach of a dead cat, and retained it there for a few minutes, a similar alteration of the texture of the internal membrane took place, but it assumed a lighter grey color. The difference of color may be explained by the vessels in the one case being empty, and in the other case being distended with blood, at the time of the injection being made.

The destruction of the substance of the internal membrane of the stomach, precludes the idea of the poison having been absorbed into the circulation. We must conclude that death was the consequence of the chemical action of the poison on the stomach. This organ, however, is not directly necessary to life, since its functions, under certain circumstances, are suspended for hours, or even for days, without death being produced. Although the stomach was the part primarily affected, the immediate cause of death must be looked for in the cessation of the functions of one or more of those organs, whose constant action is necessary to life. From the scarlet color of the blood in the left side of the heart, in the experiment on the rabbit, we may conclude that the functions of the lungs were not affected; but the affection of the earth and brain is proved by the convulsions, the insensibility, the affection of the pulse in both experiments, and the sudden cessation of the heart's action in the first, and we may therefore be justified in concluding, that the immediate cause of death death was in both of these organs. As the effects produced appear to have been independent of absorption, we may presume that the heart, as well as the brain, was acted on through the medium of the nerves.

That a sudden and violent injury of the stomach should be capable of thus speedily proving fatal, is not surprising when we consider the powerful sympathy between it and the organs, on which life more immediately depends, and the existence of which many circumstances in disease daily demonstrate to us.

VII. The facts which have been stated, appear to lead to the following conclusions respecting the action of the mineral poisons which were employed in the foregoing experiments.

1. Arsenic, the emetic tartar, and the muriate of barytes, do not produce their deleterious effects until they have passed into the circulation.

2. All of these poisons occasion disorder of the functions of the heart, brain, and alimentary canal; but they do not all affect these organs in the same relative degree.

3. Arsenic operates on the alimentary canal in a greater degree than either the emetic tartar or the muriate of barytes. The heart is affected more by arsenic than by the emetic tartar, and more by this last than by the muriate of barytes.

4. The corrosive sublimate, when taken internally in large quantity, occasions death by acting chemically on the mucous membrane of the stomach, so as to destroy its texture; the organs more immediately necessary to life being affected in consequence of their sympathy with the stomach.

In making the comparison between them, we observe that the effects of mineral are less simple than those of the generality of vegetable poisons; and, when once an animal is affected by the former, there is much less chance of his recovery than when he his affected by the latter.

---

*The License of Dr. Sheldon, Archbishop of Canterbury, granted to William Lilly the Astrologer, to practise Physic.*

The noted WILLIAM LILLY the *Astrologer*, in the latter part of his life, was a practitioner in physic: and, that he might pursue this vocation without let or molestation, through the intervention of Elias Ashmole, and under the testimonial, as to competency, of two doctors of the College of London, he procured the license of Dr. Sheldon, the then archbishop of Canterbury. This license, dated 1670, ran in the following terms:

GILBERTUS providentia divina Cantuariensis Archiepiscopus totius Angliæ Primas et Metropolitanus, dilecto nobis in Christo *Gulielmo*

*Lilly* in Medicinis Professori, salutem, gratiam, et benedictionem. Cum ex fide digna relatione acceperimus te in arte sive facultate Medicinæ per non modicum tempus versatum fuisse, multisque de salute et sanitate corporis verè desperatis (Deo Omnipotente adjuvante) subvenisse, eosq.; sanasse, nec non in arte predictà multorum peritorum laudabili testimonio pro experientia, fidelitate, diligentia et industria tuis circacuras quas susceperis peragendas in hujusmodi Arte Medicinæ merito commendatum esse, ad practicandum, igitur et exercendum dictam Artem Medicinæ in et per totam Provinciam nostram Cant. *(Civitate Lond' et circuitu septem milliarum eidem prox' adjacen' tantummodo exceptis)* ex causis prædictis et aliis nos in hoc per te justè moventibus, præstito primitus per te Juramento de agnoscendo Regiam supremam potestatem in causis ecclesiasticis & temporalibus ac de renunciando, refutando, & recusando omni, & omni modo Jurisdictioni, Potestati, Authoritati, & Superioritati foraneis juxta vim formam & effectum Statui Parlamenti hujus Inclyti Regni Angliæ in ea parte editi et provisi quantum nobis per Statuta hujus Regni Angliæ liceat et non aliter neque alio modo te admittimus & approbamus Tibiq; Licentiam et Facultatem nostras in hâc parte, Tenore præsentium quamdiu te benè & laudabiliter gesseris benignè concedimus & elargimur. In cujus rei Testimonium Sigillum (quo in hac parte utimur) præsentibus apponi fecimus. Dat. Undecimo Die Mensis Octobris, Anno Domini 1670. Nostræque Translationis Anno Octavo.

Sigillum

Radulph Snowe }<br>
et } Registrarii.<br>
Edm. Sherman }<br>
S. Rich. Lloyd, Sur.

Vicarii in Spiritualibus Generalis per provinciam Cantuariensem.

It does not appear, in the Memoirs of Lilly, as written by himself, that he ever made an attempt to acquire the elements of medical science, but was directed in his prescriptions by his astrological art only; but, having procured the above license, "he began to practise more openly; and every Saturday rode to Kingston, where the poorer sort flockt to him from several parts, and received much benefit by his prescriptions, which he gave them freely and without money. From those that were more able, he now and then received a *shilling*, and sometimes an *half-crown*, if they offered it to him, otherwise he demanded nothing."

---

*Observations on the Preparation of Soporific Medicines from common Garden Lettuce. (Lactuca sativa, Linn.) By* Andrew Duncan, Sen. M.D.—March 6, 1810.

Opium, or the inspissated white juice which exudes from the capsule of the *papaver somniferum*, when wounded, has long been allowed to be one of the most useful articles employed in the alleviation or cure of diseases. The high encomium bestowed upon it by the illustrious Sydenham,* has been fully confirmed by the testimony of many succeeding practitioners. It is, however, much to be regretted, that there are individuals of the human species, with whom, from peculiarity of habit, opium seldom fails to produce distressing consequences. There are also conditions of disease, in which it may be very necessary to induce sleep, or allay pain, though circumstances occur by which the use of opium at that time is contraindicated. Hence, it has been long a desideratum in the healing art, to discover other powerful quieting medicines. For, although it is hardly to be expected, that an article will ever be discovered so extensively useful as opium, yet, a good soporific may be found, which, with some, will have less influence, either as exciting sickness at stomach, as occasioning confusion of head, or as inducing a state of constipation.

It has been the opinion of many, that all the milky juices spontaneously exuding from wounded vegetables, possess somewhat of the same sedative power, with the milky juice of the poppy. Few plants in Britain afford such milky juice more copiously than the common garden lettuce, the *Lactuca sativa* of Linnæus; and every one must have observed, that this juice, when spontaneously inspissated by the heat of the sun, on the wounded plant, soon assumes the dark color of opium, while, at the same time, it possesses in a high degree the peculiar, and I may say specific, taste, which distinguishes that substance. And besides this, it is a well-known fact, that lettuce was much used by the ancients as a soporific.

These circumstances led me to turn my thoughts on some method of collecting and preparing this substance, that I might try its effects in the practice of medicine. And, after several trials of different modes of preparation, what I shall now briefly describe are the best methods I have yet been able to discover.

I dedicated to this experiment, in my garden at St. Leonard's-hill, near Edinburgh, a small bed of that variety of lettuce, which is commonly known among gardeners by the name of *ice-lettuce*. I allowed the plants, about a hundred in number, to shoot up, till the top of the stem was about a foot above the surface of the ground. I then cut off about

---

* "Ita necessarium est Opium, in hominis periti manu, ut sine illo, manca sit, ac claudicet medicina."—*Sydenham, de Dysenteria anni* 1670, *&c.*

an inch from the top of each. The milky juice immediately began to rise above the wounded surface. Though then of a white appearance, it had next day formed a black, or dark-colored incrustation, over the surface where the stem was cut off. I found it impossible to separate this by scraping, as is done by the milky juice exuding from the head of the poppy, when it has assumed the form of opium. I therefore cut off with a sharp knife a thin cross slice of the stem, to which the whole of the dark-colored opium-like matter adhered. This was thrown into a wide-mouthed phial, about half filled with weak spirit of wine, the *alcohol dilutum* of the Edinburgh Pharmacopœia, formed of equal parts of rectified spirit and water. By this menstruum, the whole black incrustation on the thin slice of the stalk was dissolved, and the spirit, as may readily be concluded, obtained both the color and taste of the black incrustation.

Each of my plants, in consequence of the fresh wound inflicted by the removal of the thin cross slice, afforded a fresh incrustation every day. And, by throwing these into the phial, I soon obtained what I concluded to be a saturated solution of the exudation from the lettuce, or rather of the milky juice in its inspissated state. It was then strained off, to separate the pure solution completely from the thin slices of the stalk. To this strained spirit, which had nearly both the appearance and taste of the ordinary laudanum of the shops, I have given the name of *Solutio spirituosa succi spissati lactucæ*. From trials made with this solution, both on myself and others, I have no doubt that it is a powerful soporific. But to obtain a form in which it might be exhibited, with greater certainty as to the dose, I evaporated the spirit, and thus brought the residuum to a dry state. In this state, it has very much the appearance of the opium imported into Britain, particularly of that which is imported from Bengal, and which is a much softer substance than the Turkey opium. To this opium-like substance, I have given the name of *Lactucarium*. And, from some trials which I have made with it, when exhibited under the form of pills, it appears to me to be little inferior in soporific power to the opium which is brought from Bengal.

From the *lactucarium* thus obtained, I have formed a tincture, by dissolving it to the extent of one ounce, in twelve of weak spirit, which is the proportion of opium to spirit, in the liquid laudanum of the Edinburgh College. To this formula, I have given the name of *Tinctura lactucarii*. I consider it as the best formula I have yet been able to contrive, for obtaining the soporific and sedative powers of the lactuca sativa. And in different cases, I have, I think, seen manifest manifest good effects from it, both as inducing sleep, allaying muscular action, and alleviating pain, the three great qualities of opium, which demonstrate it to be one of the most powerful sedatives. At present, however, I intend nothing more but to communicate to the Caledonian Horticultural Society a method of preparing a soporific medicine from common lettuce. For ascertaining more fully its medicinal effects, I am at present engaged in a series of trials, which may perhaps be likewise communicated to them.

Meanwhile, it will afford me great satisfaction, if the above short account shall draw the attention of others, particularly of professional gardeners, to the same subject, and shall lead to the discovery of a better method of obtaining an useful medicine, from a plant so easily cultivated in every garden. Perhaps this important object might be somewhat forwarded, if the Caledonian Horticultural Society were to propose a prize, as a reward to the person who should be most successful in preparing a medicine from the milky juice of the Lactuca. But it should be an essential condition of that prize, that he should send them not only a specimen of the substance prepared, but also an exact account of his method of preparing it.

In consequence of the above suggestion, the Caledonian Horticultural Society, at their quarterly meeting, on the 6th of March, 1810, agreed to propose a Prize Medal for each of the two following questions:

1. For the best method of preparing a soporific medicine from the inspissated white juice of the common Garden Lettuce. Specimens of the medicine to be produced.

2. For the best method of preparing opium in Britain, and the most advantageous manner of cultivating poppies for that purpose.

*Further Observations on the Preparation of a Soporific Medicine from common Garden Lettuce. By* Dr. Duncan, sen. November, 1811.—From the writings of the most eminent medical authors, it appears that garden lettuce was employed many centuries ago, for the purpose of procuring sleep. Galen, who flourished about the commencement of the Christian era, mentions it frequently in his writings. And it is said, that in an advanced period of life, when distressed for want of sleep, he used it with success.*

* "Hypnoticam esse jam cognoverunt veteres: Celsus, qui papaveri ideo adjungit; Galenus, qui sibi ipsi senex insomnis vesperi lactuca comesa somnum conciliavit."—*Murray, Apparatus Medic.* vol. i. p. 109.

Among

Among the moderns, this article has not been altogether neglected. Some observations and experiments have been made respecting its medical powers, both in England and in America, particularly by Dr. Cox, of Philadelphia, and Dr. George Pearson, of London.

About two years ago, I read to the Caledonian Horticultural Society, a short account of a method of preparing a soporific medicine from this plant. That account so far engaged the attention of that Society, that they proposed a Prize Medal, as an honorary reward, *for the best method of preparing a soporific medicine from the inspissated white juice of the common garden lettuce.*

I am happy to learn that some ingenious men have not been neglectful of this subject; and I would fain hope, that even our inconsiderable premium might lead to an honorable and useful competition. Among others, I have myself made farther trials with this vegetable, and I now present to the Society specimens of five different preparations of lettuce, all of which may, I think, be usefully employed in the practice of medicine.

Of the method of preparing the first, second, and third, of these, viz. 1. The Spirituous Solution, or tincture of the dried juice; 2. The Extract, which I formerly styled *Lactucarium*, and which is prepared by the evaporation of that solution or tincture; and, 3. The Tincture of the Lactucarium, which is prepared by dissolving that substance in diluted spirit of wine, I have nothing to add to what I formerly related to the Society. I may however observe, that, from repeated trials, I have found all of them to be useful soporifics. But the preparation of these requires much time and great attention; and, in preparing the lactucarium, it may be easily injured by the improper application of heat.

The two additional preparations, which I now present to the Society, the Inspissated Juice, and the Tincture of the leaves of Lettuce, may be made very easily, and at a very trifling expence. Although not so powerful as the solution or extract, prepared from the inspissated milky juice, yet they will, I am persuaded, be found upon trial, to be highly useful in the practice of medicine.

*Method of preparing the Inspissated Juice of Lettuce*, *or the Succus Spissatus Lactucæ recentis.*—Take any quantity of the leaves and stalks of the lettuce, when the plant is nearly ready to flower; bruise them well, and, including them in a hempen bag, compress them strongly till they yield their juice. Let this juice be evaporated in flat vessels, heated with boiling water. Let the evaporation be continued till the expressed juice be reduced to the consistence of thick honey.

According

According to the trials which I have made, twelve pounds of lettuce will yield about eight ounces of inspissated juice.

*Method of preparing the Tincture of Lettuce-leaves; or the Tinctura foliorum siccatorum Lactucæ sativæ.*—To one ounce of the dried leaves and stalks of the lettuce cut down, add eight ounces of the diluted alcohol of the Edinburgh Pharmacopœia. Let the vessel containing this mixture, be kept for a week in a warm place, shaking it frequently. Let the liquor then be strained through paper, and kept for use. About fifty drops may be taken for a dose.

*Additional Observations on the Lactuca, presented to the Caledonian Horticultural Society, by* Dr. Duncan, sen.—May 1, 1812.—In two former short communications to the Society, I have given an account of a method of preparing from the common garden lettuce different articles, which may, I am convinced, be employed with advantage in the practice of medicine. To these I have given the following names:

1. *Solutio vel Tinctura succi spissati Lactucæ*, prepared from the inspissated juice spontaneously exuding from the plant when wounded.

2. *Lactucarium*, an extract prepared by evaporating the above solution or tincture.

3. *Tinctura Lactucarii*, prepared by dissolving the lactucarium in proof-spirit of wine.

4. *Succus spissatus Lactucæ*, prepared by inspissating the expressed juice of the recent plant.

5. *Tinctura foliorum Lactucæ*, prepared by extracting the active powers of the lettuce, from the leaves of the dried plant, by warm infusion in proof-spirit.

To my former observations I can now add, that, during the course of last winter, I have made many trials of these articles, both in hospital and in private practice. I have particularly employed the first and the fourth of these preparations, in the clinical wards of the Royal Infirmary, where the effects were observed by many attentive and ingenious students. They have witnessed the benefit which may be derived from them in procuring sleep, in alleviating pain, and in allaying inordinate action, particularly troublesome cough. I am therefore not without hopes, that when the experiments I have made are more generally known, they may have the effect of calling the attention of other medical practitioners, and of some intelligent gardeners, to a subject, which, in my opinion, is of considerable importance.—*Memoirs of the Caledonian Horticultural Society.*

On

*On Vegetable Wax, &c. By* R. MACCULLOCH, M.D. Woolwich.

IT is now well known that wax is a vegetable product, as well as the result of an animal process in bees and other insects, and the wax of various plants has been successively examined by different chemists. Some slight differences have been observed in the several varieties, but they are not sufficient to lead us to consider them as different species; rather, like the generality of the resins, to be varieties of one common substance. To those already described there is still to be added one, which, as far as I know, has not yet been noticed. This is a substance held in solution in the essential oil of the rose (the attar of roses) and in that of lavender. I have not searched among the other oils, but it will probably be found in some of them. All the varieties of these two oils do not however contain it; it is frequently absent in the oil of lavender, although but rarely in that of the rose.

I am not acquainted with the circumstances under which this variation occurs. When these oils are cooled below a certain point, a portion of this matter is deposited in the form of minute crystals, giving them an appearance somewhat similar to that which the fixed oils assume on freezing. On the addition also of alcohol it is separated in the form of minute brilliant scales, and by this method I obtained the portion which I examined. It is equally separated by water, which, if enough be used, dissolves the whole of the oil, and leaves it in a pure state. It is thus that it is collected in the pipes of the stills in which rose-water is made, as it is volatilized in combination with the oil, and precipitated by the action of the water which is condensed in the worm. That with which I made the following experiments was procured from lavender; but it seemed to differ in no respect from that which I have procured from the oriental attar of roses, or from the distillation of rose-water.

Although I have called it wax in consideration of its vegetable origin, it bears in fact a much nearer resemblance to spermaceti in its general properties. Like that, its feel is greasy, and it is deposited in a crystallized mass at the bottom of the vessel, just as that substance is deposited from the oil of the Cachalot whale.

The few comparative experiments which follow, will show its nature more completely. Having but a very small quantity, I could not conveniently determine its specific gravity; but it is much lighter than either wax or spermaceti, since it swims in sulphuric ether. It crystallizes from its solutions in resplendent scales, and in this property it approaches rather to spermaceti than wax. Its color is white, and its texture flaky.

flaky. It is fusible in 96°, while wax is only fusible at 120°, and spermaceti at 102°. This account of the fusibilities of wax and spermaceti differing from that commonly received, which states them at 142° and 133° respectively, it is necessary to say that the mode which I took to determine this temperature, and to which I was compelled by the scantiness of my materials, was by causing them to melt on hot water in which a thermometer was immersed, and noting the heat at the moment of congelation. In boiling alcohol it dissolves readily and in as large proportion as spermaceti, more readily and in larger proportion than wax; and it is deposited again on cooling. The three substances seemed equally soluble in boiling ether, which however dissolves less of them than alcohol does. Its habits with the other compound inflammables, and with the alkalies, resemble those of wax and spermaceti, and afford no distinction.

It is volatilized without apparent change in a temperature considerably lower than spermaceti, and I need not add, that its vapor is equally inflammable. I had no adipocire with which to compare it.

Considering these circumstances, we may perhaps regard it as a vegetable concrete oil, resembling spermaceti rather than wax, yet differing from it in the characteristic circumstances of superior volatility and inferior specific gravity, and bearing a relation to essential oils similar to that which spermaceti does to the fat ones.—*(Phil. Mag.)*

## Figures and Tables

**Figure f1:**