# Corrigenda

**Published:** 1812-09

**Authors:** 


### *CORRIGENDA.*

In our last, p. 128, l. 5 from bottom, for *earth* read *heart*.

